# Accommodative intraocular lenses: where are we and where we are going

**DOI:** 10.1186/s40662-017-0077-7

**Published:** 2017-06-26

**Authors:** Jorge L. Alió, Jorge L. Alió del Barrio, Alfredo Vega-Estrada

**Affiliations:** 1 Cornea, Cataract and Refractive Surgery Unit, Vissum Corporación, Avda de Denia s/n, Edificio Vissum, 03016 Alicante, Spain; 2 Division of Ophthalmology, Universidad Miguel Hernández, Alicante, Spain

**Keywords:** Accommodation, Accommodative intraocular lens, Presbyopia

## Abstract

Presbyopia still remains the last frontier of refractive surgery. Its surgical management is under constant evolution due to the limitations that exist today with respect to its management, which is probably in relation with the multifactorial basis in which presbyopia is clinically developed in the human. Until currently, virtually all surgical techniques that have been proposed for its correction are based on the induction of pseudoaccommodation in the presbyopic eye, including multifocality. However, the real restoration of accommodation is more complex, and it has been tried by the use of different, so called, “accommodative” pseudophakic intraocular lenses (AIOL). Overall, the reported results with these lenses by independent authors have been modest in relation with the restoration of the accommodative power of the eye and these modest benefits are usually lost with time due to the long term changes in the capsular bag. This fact made these lenses to be almost abandoned in the last few years, but there are currently other AIOL models being used with innovative mechanisms of action and different anatomical support outside the capsular bag that offer encouraging preliminary results that could bring a new potential of application to these types of lenses.

In this article, we will update the modern refractive surgeon about the fundamentals and provide updated information about the outcomes of AIOLs by reviewing the concept of accommodation, the different attempts that have been accomplished in the past, their demonstrated published results in human clinical trials, and the future alternatives that may arrive in the near future.

## Background

Presbyopia is the physiological degradation of accommodation and still remains as the last frontier of refractive surgery as its surgical management is perhaps the most innovative and challenging and is under constant renewal. The multifactorial basis for the development of presbyopia makes it difficult to be managed adequately.

The aging population, especially in western countries, has created millions of candidates around the world for such interventions. Current non-surgical treatment options include reading spectacles (either mono or varifocal) and contact lenses (either multifocal or monofocal for monovision), but many of these patients, spectacle independent previously, do not easily accept this unavoidable age-related life style change. While corneal procedures for presbyopia are still under a serious debate regarding their long term outcomes and success rate, current surgical options mostly include refractive lens exchange by either monofocal IOLs for monovision or multifocal IOLs. However, none of them could achieve a complete restoration of accommodation, and multifocal lenses are frequently associated to visual symptoms that may decrease patient satisfaction. Therefore, presbyopic surgery is one of the most difficult targets that a refractive surgeon will have to deal with today and in the immediate years.

Almost all surgical techniques that have been proposed to date for the surgical correction of presbyopia are based on the acquirement of pseudoaccommodation [1]. Pseudoaccommodation comprises a group of techniques that can improve near vision on the basis of the use of the cornea or intraocular lenses (IOLs), resulting in an increased depth of field, multifocality or both [2]. Pseudoaccommodation may be accomplished by the induction of certain amounts of higher order aberrations and achromatization, among other less important methods [2], and they should be studied in lenses wherein real accommodation is the target as it is often masqueraded in the outcomes of AIOLs [3]. Some of these techniques are based on the adequate performance of the ciliary body using part of the physiological accommodative mechanism, but a surgery for the “real” restoration of accommodation has not yet arrived for clinical practice.

The aim of this review article is to provide the modern refractive surgeon with updated information about this topic, review the concept of pseudophakic accommodation as well as different attempts that have been made in the past to achieve a real restoration of accommodation via the use of intraocular lenses as evidenced in human clinical trials, and finally, consider future alternatives that may present themselves in the near future.

## Accommodation: concepts and definitions

### Accommodation

The change in the refractive power of the eye, when the image of a near object is brought into focus on the retina, is defined as accommodation; such a process must involve an increase in the dioptric power of the system [4]. Helmholtz (1856) proved that accommodation is accomplished by the change of the power of the crystalline lens associated with the active action of the ciliary body.

### Amplitude of accommodation

It is the difference in refractive power of the eye in the two states of complete relaxation and maximal accommodation. The amplitude of accommodation decreases progressively with age (presbyopia): in a ten year old child it is, on average, about 14 D; at forty it is 6 D; and at 60 it is only about 1 D.

### Pseudoaccommodation

This concept refers to any other method that changes the power of the whole optical system of the eye or changes partly the way in which it works in order to relieve patients for near distance vision. Nevertheless, pseudoaccommodation is not a real restoration of accommodation [1, 5]. It can restore near distance vision by different mechanisms:

- *Changes in the axial position of an IOL inside the eye*, thereby inducing a change in the overall power of the eye. Such lenses can be considered "partially accommodative".
- *Multifocality*: multifocal optics either on the cornea (PresbiLASIK) or on an intraocular lens (diffractive or refractive).
- *Increased depth of focus* using different methods such as pinhole effects. Depth of focus is considered to be one of the most important factors of apparent pseudoaccommodation [2]. Nakazawa et al. demonstrated that the amount of pseudoaccommodation is highly correlated with the calculated depth of focus in eyes implanted with monofocal IOLs [6]. On the other hand, depth of focus is negatively correlated with normal visual acuity (the higher the visual acuity, the lower the depth of focus) [7]. This effect can be achieved by changes in the pupil size (pupillary diameter has been demonstrated to be inversely proportional to pseudoaccommodation) [8]; in the astigmatic component of the cornea (astigmatism against-the-rule offers better near visual acuity when compared to with-the-rule astigmatism, while the unaided far vision is equally affected) [9]; and also by positive or negative changes in corneal aberrations (as spherical aberration, coma-like aberration or vertical coma) [10, 11].

The theory of accommodation is largely based on Helmholz's ideas: the capsule has sufficient elasticity to mold the lens into a more strongly curved system than what is necessary for distance vision. The elasticity of the capsule is held in check by the normal tension in the zonule so that accommodation consists in the relaxation of the tension in the zonule by the contraction of the ciliary muscle. This permits the capsule to mold the lens into a more strongly curved system (Table 1).

**Table 1:** Summary of the critical changes during accommodation

*Accommodated eye*
1. Sphincter-like action of circular muscle fibers contraction
2. Contraction of ciliary muscle
3. Distance between edges of ciliary body decreases
4. Relaxation of suspensory ligament
5. Lens becomes thicker
6. Focal length shortens
*Unaccommodated eye*
1. The ciliary muscle is relaxed
2. Aqueous and vitreous humor push outward on the sclerotic coat
3. Ligaments become taut/tensed
4. Lens pulled into a thin shape
5. Focal length becomes short

According to Fincham’s findings where the curvature of an excised senile lens is considerably less than that from a juvenile one [12], the loss of accommodative power (presbyopia) is essentially due to the progressive failure of the capsule to mold the lens into a more spherical shape when the zonule is relaxed as a consequence of the progressive sclerosis and hardening of the lens substance with age. Nevertheless, it has been demonstrated that the anterior zonular attachment on the equatorial edge of the lens shifts forward with age [13], and these changes in the configuration of the ciliary muscle may reduce the ability of the zonular fibers to release tension on the lens during accommodation thereby playing a role in the development of presbyopia as well. It is likely that the ultimate mechanism for presbyopia is a culmination of many factors together resulting in a loss of accommodative amplitude (multifactorial theory). However, it is unclear if these documented changes in the ciliary muscle and the lens sclerosis occur together or if one is a consequence of the other.

## Accommodative IOLs: basic principles

A truly accommodative IOL (AIOL) would be the one capable of undergoing a progressive change in its power in relation with the active contraction of the ciliary body [1]. Thus, the ideal AIOL would fully resolve the inconvenience of presbyopia and the side effects in relation with current surgical options as the positive visual symptoms or the deterioration of quality of vision after multifocal IOL implantation. Obviously, this ideal AIOL would have a huge impact in refractive surgery and private practice, which explains the interest from the different companies in developing such lenses. This ambition has led to many mistakes in the past (commercial bias, poor methodology to study near vision, non-independent monitorization, etc.), where different types of AIOLs were presented to the scientific community as highly effective to be then discredited later on by independent studies from different authors.

After multiple failures, the question arises if AIOLs replicating the mechanism of accommodation could actually be developed. Abundant research is still necessary in order to answer this question, but we could at the very least learn from past mistakes, which gave rise to some clear some concepts [3]:

- Outcomes should be tested by homologated charts for near (40 cm) and intermediate (70 cm) vision
- Accommodation should be measured by subjective and objective tests
- Pseudoaccommodation needs to be identified in the outcomes
- Results have to be confirmed by multicentric series and in long term study observations

To date, three basic approaches for AIOLs have been accomplished:

- Change in axial position
  - ◦ Single or dual optic
- Change in shape or curvature
- Change in refractive index or power

The majority of AIOLs that have been proposed are not really accommodative lenses as their mechanism of action is based on changing the axial position of a monofocal IOL in relation with the cornea, hence changing the global eye power. However, there is not a real change in the IOL power itself. Theoretically, when plate lenses are placed into the capsular bag, the anterior capsule fibroses and applies end-to-end pressure on the plates, which vaults posteriorly and the optic comes to lie up against the vitreous face. When the ciliary muscle constricts, it redistributes its mass like any other muscle and encroaches on the vitreous cavity space, increasing the vitreous cavity pressure, moving the optic forward. Approximately, 1 mm of movement is equivalent to almost a 2 D power change.

These lenses are known as “positional pseudoaccommodative IOLs”, and their visual results in terms of providing partial or total near visual acuity restoration in the long term have been disappointing.

The aim of this report is to provide an update and a general overview for the anterior segment ophthalmologist regarding the current state of the art AIOLs that have already been the subject of human clinical trials with evidence available by peer reviewed scientific publications.

## Review

A PubMed review was performed, analyzing all publications from 2005 to 2016 concerning the topic “accommodative pseudophakic intraocular lenses” (keywords: accommodation, intraocular pseudophakic lenses, accommodative intraocular lenses). Only published studies (in English – full text) about accommodative lenses implanted in human clinical trials and with evidence level Grade I or II were included for the purpose of this publication. The following were the accommodative intraocular lenses reported in the literature review (Table 2):

**Table 2:** Accommodative intraocular lenses main features. Hydroxyethylmethacrylate (HEMA), intraocular lens (IOL)

	Crystalens	1CU Lens	Tetraflex	Synchrony	Lumina	Nulens	WIOL-CF
Material	Silicone	Hydrophilic acrylic material	Hema	Silicone	Acrylic	PMMA/SILICONE	Methacrylic copolymer
Location	Capsular bag	Capsular bag	Capsular bag	Capsular bag	Ciliary sulcus	Ciliary sulcus	Capsular bag
Mechanism of Action	Single optic-forward motion	Single optic-forward motion	Single optic-forward motion	Dual optic IOL	Alvarez Principle	Axial motion	Axial motion
Objective accomodation	<0.4 D [8]	no [14–16]	2 D [17, 20]	*	2-3 D	*	*
Evidence of pseudoaccommodation	yes [8–10]	yes [14–16]	yes [19, 20]	*	*	*	*
Commercially available	Yes	Yes	Yes	Yes	No	No	No

*not well reported according to published literature

### Crystalens

**Fig. 1:**
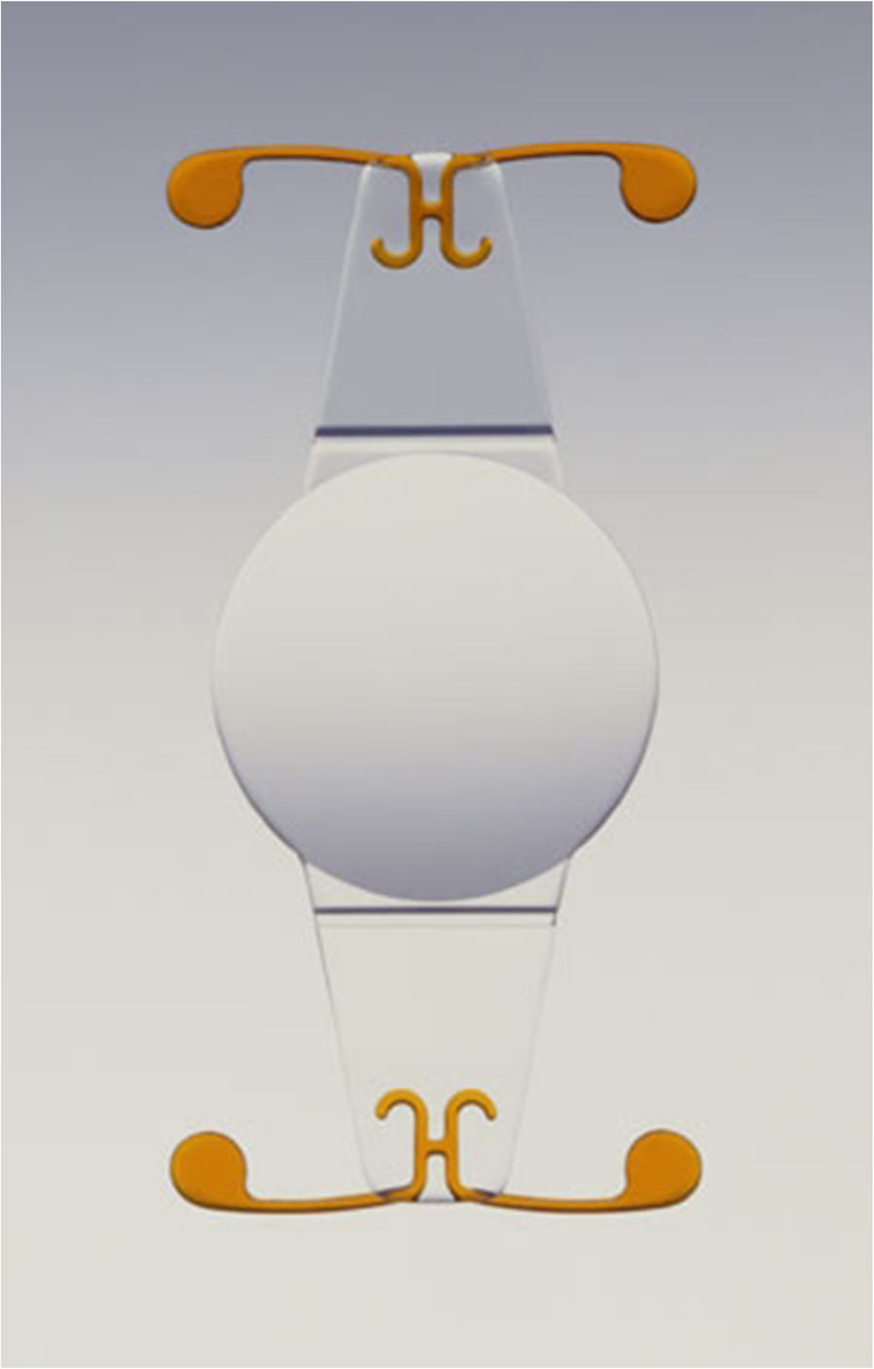
Crystalens AIOL. Reprinted with permission from [19].

Eyeonics Crystalens (Eyeonics, Inc., Aliso Viejo, CA, USA) is manufactured from high-refractive-index silicone material containing an ultraviolet (UV) filter. To decrease the resistance of the optic to forward motion, the lens incorporates hinges adjacent to the optic across the plates (Fig. 1). Fixation within the capsular bag is ensured by the presence of small, T­shaped haptics at the end of the plates.

**Fig. 2:**
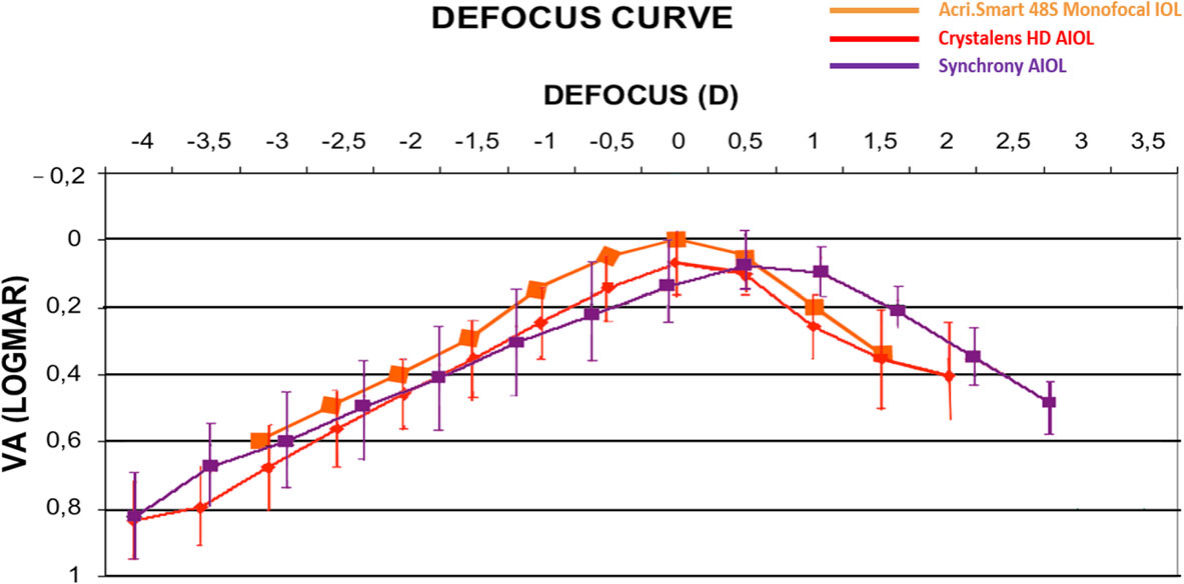
Median defocus curve by group. The error bars represent the range associated with each median value (VA: visual acuity; IOL: intraocular lens; AIOL: accommodative intraocular lens). Reprinted with permission from[20]

There seems to be an agreement among authors that distance visual acuity results with Crystalens AIOLs do not differ from those obtained with monofocal IOLs. However, contradictory data can be found regarding intermediate and near visual acuities. Despite some authors still reporting significantly improved intermediate and near visual results in comparison with monofocal IOLs [14], the majority of them report very poor results [15–20]. Vilupuru at al. reported poor distance-corrected near visual acuity (DCNVA) results in comparison with Restor +3 D multifocal IOL (mean LogMAR: 0.360 versus -0.042, respectively), obtaining slightly better results for distance-corrected intermediate visual acuity (DCIVA) (mean logMAR: 0.186) [15]. The accommodative response measured objectively using laser ray tracing aberrometry has been reported to be lower than 0.4 D with this lens [16]. In this study, the authors also observed changes in astigmatism, spherical aberration, trefoil, and coma with accommodation in the Crystalens AIOL group, which should arise from geometrical and alignment changes in the lens with accommodative demand. Therefore, pseudoaccommodation from increased depth of focus may justify the moderate benefits for DCIVA and mild reported changes in DCNVA [17, 18]. Our group has also demonstrated in different papers the poor defocus curve shown by this type of AIOL (Fig. 2), whereas a multifocal IOL (Lentis-Mplus) showed significantly better visual acuities at several defocus levels. On the other hand, the Crystalens group showed better contrast sensitivity under photopic conditions at all spatial frequencies [19, 20].

### AG Akkommodative 1CU lens

The Akkommodative lCU lens (HumanOptics AG, Erlangen, Germany) is made of a hydrophilic acrylic material. The principle action of this lens is based on the anterior movement of the optic secondary to the ciliary muscle contraction. The haptics of the lens are modified with transmission elements at their fusion with the optic.

The accommodative properties of this lens are very dependent on the flexibility of the capsular bag, which was what made this lens fail in the long term due to the unavoidable contraction of the capsule [21]. Mastropasqua et al reported a complete loss of the 1CU AIOL accommodative properties within 2 years (DCNVA of 8.1 Jaeger at 1 year postop) because of the high incidence and degree of anterior and posterior capsule opacification (100% of patients after 1 year), probably induced by the lens material and design themselves [21]. Other authors have found a minor improvement in the near visual function compared with monofocal lenses, but have not found any evidence of measured accommodative amplitude. Therefore, these changes are likely to be explained by pseudophakic pseudoaccommodation in a fashion similar to the Crystalens AIOL [22–24].

### Kellen Tetraflex accommodating lens

The Tetraflex KH-3500 (Lenstec Inc, FL, USA) is a one-piece highly flexible hydroxyethylmethacrylate (HEMA) lens. The lens haptic was designed to take advantage of how the crystalline lens moves during accommodation according to the Helmholtz theory. It is not based on a hinge principle, but rather on a haptic configuration to allow the lens to move with the entire capsular bag

The initial sponsored publications reported good results with 75% of patients with at least 2 D of accommodative amplitude 6 months after surgery and a better near visual function compared with the Crystalens AIOL [25, 26]. There seems to be an agreement that Tetraflex enhances near visual function compared with monofocal IOLs, but it has been demonstrated that the Tetraflex AIOL actually is relatively fixed in position within the eye. Therefore, some of these reported benefits appear to be in relation with changes in the optical aberrations because of the flexure of the IOL on accommodative effort rather than forward movement of the lens within the capsular bag [27, 28]. Nevertheless, the results are still far from those obtained with multifocal pseudoaccommodative IOLs, and independent studies were not able to demonstrate significant differences in near and intermediate vision compared with mini-monovision with monofocal lenses or even Crystalens AIOL [29, 30]. A final concern raised with this lens was its vulnerability to the contraction of the capsular bag due to its highly flexible hydrophilic acrylic material, with a subsequent anterior flexing of the lens haptic component requiring the exchange of the AIOL in many cases according to the authors of this report, personal experience and isolated reported case reports [31].

### Synchrony dual optics IOL

**Fig. 3:**
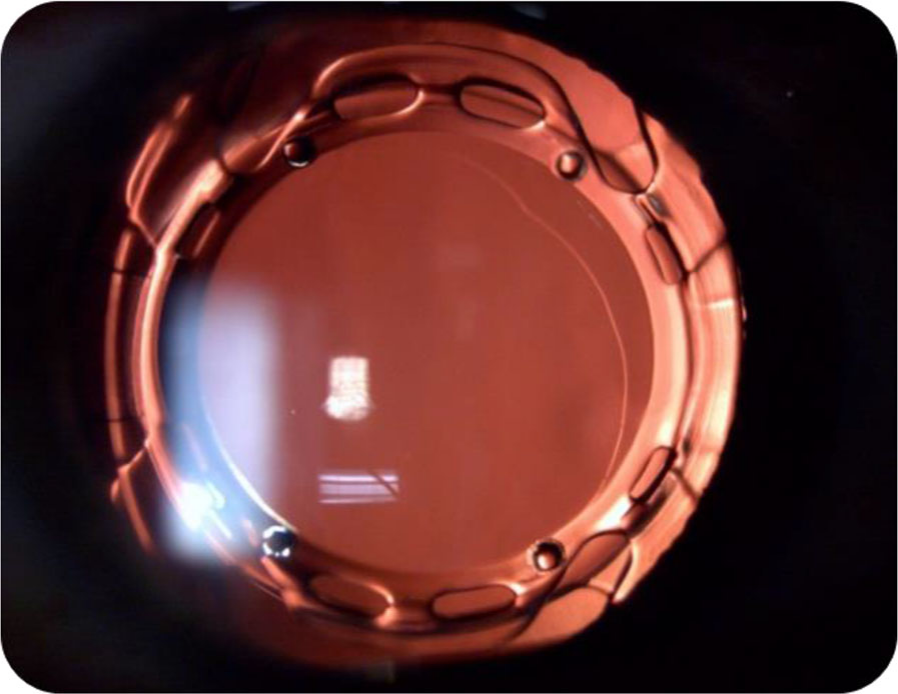
1CU AIOL

Synchrony AIOL (Visiogen, Inc.) is a dual-optic silicone lens. It has 2 main components (anterior and posterior): each component has the general design of a plate haptic silicone IOL, with a bridge between them with a spring function connecting the 2 components (Fig. 3). The anterior IOL component has a high plus power beyond that is required to produce emmetropia. The posterior IOL component has a minus power to return the eye to emmetropia. Once the IOL is in the capsular bag, the tension of the bag compresses the optics. During accommodation, the contraction of the ciliary body causes zonular relaxation, which releases the tension on the capsular bag and in consequence releases the spring that increases the interoptical distance and also the IOL power. The posterior lens is designed with a significant large area to reduce the tendency toward posterior axial excursion and to maintain stability and centration within the capsular bag at all times.

Very little evidence regarding the long term outcomes of this AIOL by independent authors is currently available. Our group already demonstrated that although Synchrony showed significantly better visual acuities at several levels of defocus compared with Crystalens, as well or better optical quality and near visual outcomes were still limited [20].

Nowadays, a controversial topic is whether an AIOL should be placed inside (the classic approach) or outside the capsular bag [32]. The capsular bag is the basal membrane of the lens epithelium, and once it is emptied its fibrosis and atrophy are unavoidable as it has no function to accomplish and no anatomic structure to support. Thus, the capsular bag cannot function in the long term when it is emptied [32]. This fact has been demonstrated by a recent study performed by our group. We observed, in a primate model, that following phacoemulsification with insertion of a force/movement gauge simulating an accommodating intraocular lens, capsular fibrosis causes the disappearance of the mechanical forces detected by the in-the-bag gauges. However, the on-the-bag gauges placed at the sulcus detected stronger active forces lasting at least 5 years, although in the long term the contracting capsule pressure compromises its compliance [32]. Summarizing, the ciliary body is still active even in advanced senility, and centripetal and centrifugal forces have been demonstrated to exist in the zonular-capsular bag complex following phacoemulsification [33, 34]. However, considering the unavoidable atrophy of the capsular bag, which seems to be a wrong destination for an AIOL as it has already been demonstrated by the constant failures of the AIOL models tested to date. In this scenario, the forces generated at the zonular-anterior capsule system are probably those to be used by AIOLs, and the sulcus location may be the ideal one for such purpose. Pallikaris et al. reported, incidentally, better near vision results in a small group of eyes (*n* = 3) where a Crystalens AIOL was implanted in the sulcus, after a posterior capsule rupture, compared with the fellow eye containing this AIOL within the capsular bag. This incidental finding would be justified by the optimized forces present in the sulcus [35].

## A new generation of accommodative IOLs

Over the last few years, several approaches have been proposed in order to improve the designs and the outcomes of accommodative intraocular lenses (AIOL). Most of the AIOLs described in the previous section based their mechanism of action in the axial movement of the optics. Even when accommodation may be achieved by these AIOLs, the main limitation of this design is that it is very dependent on the adequate function of the capsular bag. As we are all aware, fibrosis and contraction of the capsular bag will eventually develop after cataract removal, thus, AIOLs that are placed in this location progressively lose the capability of restoring the accommodation of the patient. This was the main reason to develop an AIOL that will be placed in other areas different to the capsular bag, such as the ciliary sulcus where it can also benefit from the forces of the ciliary muscle. Other designs that combine different mechanism of action have also been developed, all of them trying to mimic the accommodation process of the crystalline lens.

In the following section we will summarize the main features and the published clinical results of the latest AIOL designs implanted in human eyes in clinical studies published in peer reviewed ophthalmic journals (Table 2).

### Lumina AIOL

#### The lens

**Fig. 4:**
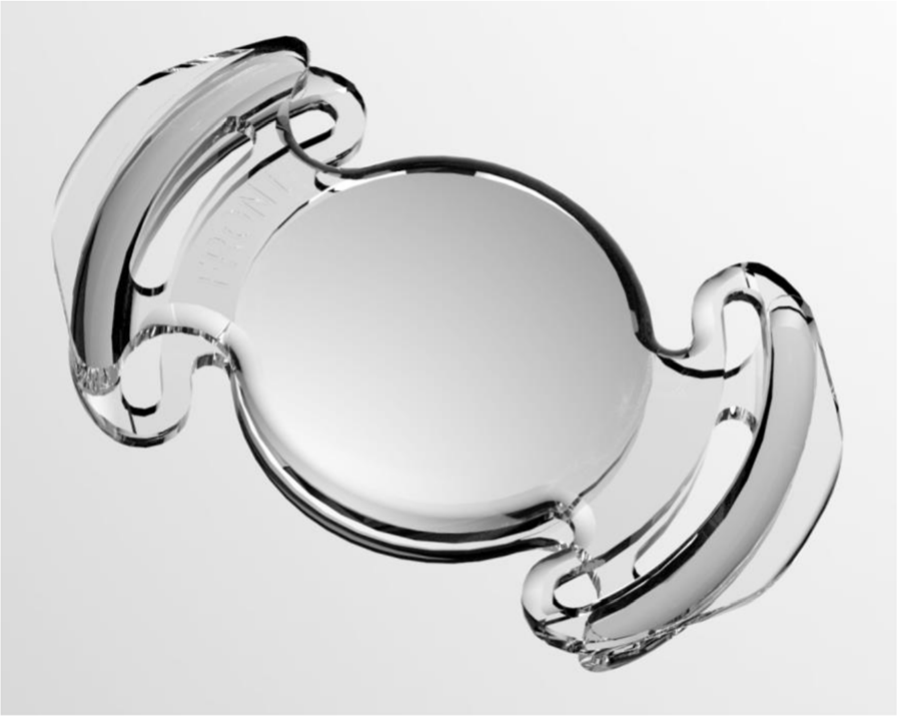
Lumina AIOL. Courtesy of Mr. Aleksey Simonov, Akkolens International b.v. Breda, The Netherlands

The Lumina AIOL (AkkoLens International, Breda, The Netherlands) consists of 2 optical elements, capable of moving one on top of the other, and is implanted in the ciliary sulcus (Fig. 4). The lens is manufactured with an acrylic hydrophilic polymer material. The optics provides a fixed optical power: the anterior element is designed to provide 5 D while the posterior provides between 10 to 25 D, depending on the correction needed for the patient after the lens removal. Each one of the optics has an internal aspheric surface where its power increases linearly when the lens moves. Therefore, when the eye accommodates and the ciliary muscle contracts, the optics of the lens change their longitudinal position, passing one over the other thereby resulting in an increase of the dioptric power of the lens, focusing the light for the near distance and providing accommodation to the patient.

The size of the IOL is personalized based on the sulcus-to-sulcus measurement of each patient. The surgical technique for the IOL implantation is similar to a standard cataract surgery procedure differing only in the placement of the IOL i.e., in the ciliary sulcus. The IOL can be implanted through a corneal incision between 2.8 and 3.0 mm.

#### Clinical outcomes after Lumina IOL implantation

Recently, our research team conducted an investigation to evaluate the first clinical results of patients implanted with this type of IOL [36]. In the study, a total of 61 eyes implanted with the Lumina AIOL were assessed during a follow up period of one year. A significant improvement in both distance and near vision was observed after AIOL implantation. Table 3 summarizes the visual and refractive results found in the study. In addition, when compared with a monofocal IOL, the Lumina AIOL also showed significantly better results in terms of uncorrected near and distance corrected near visual acuity (*p* <0.01) [36]. After 1 year of follow up, more than 90% of those patients implanted with the Lumina AIOL showed a distance corrected near visual acuity of 0.8 in the decimal scale with 70% of the patients having a spherical equivalent of ± 1 D [36].

**Table 3:** Comparative table showing the postoperative data of patients included in the Lumina intraocular lens group and the monofocal control group

Mean (SD) Range	Lumina intraocular lens (*N* = 61)	Monofocal control lens (*N* = 25)	*P*-value
LogMAR UDVA	0.24 (0.36) -0.08 to 1.40	0.06 (0.11) -0.08 to 0.30	0.21
Sphere (D)	-0.27 (1.10) -4.75 to +2.00	+0.52 (0.81) -1.25 to +1.50	<0.01
Cylinder (D)	-1.39 (0.79) -4.25 to -0.25	-1.02 (0.60) -2.00 to 0.00	0.17
LogMAR CDVA	0.05 (0.26) -0.08 to 1.40	0.00 (0.06) -0.08 to 0.10	0.73
LogRAD UNVA	0.13 (0.14) 0.00 to 0.52	0.35 (0.16) 0.00 to 0.52	<0.01
LogRAD CDNVA	0.12 (0.20) -0.08 to 1.00	0.37 (0.18) 0.10 to 0.52	<0.01
LogRAD CNVA	0.02 (0.08) -0.08 to 0.30	0.06 (0.13) -0.08 to 0.40	0.51

*SD* = standard deviation, *D* = diopters, *UDVA* = uncorrected distance visual acuity, *CDVA* = corrected distance visual acuity, *UNVA* = uncorrected near visual acuity, *CDNVA* = corrected-distance near visual acuity, *CNVA* = corrected near visual acuity, *N* = number of cases

**Fig. 5:**
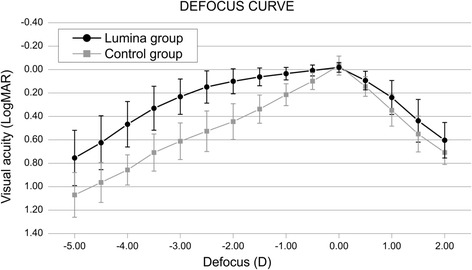
Defocus curve obtained after implanting the Lumina AIOL. Reprinted with permission from [36]

In our study, the defocus curve for both monofocal and accommodative IOL was also evaluated. It was found that the Lumina IOL provides significantly better vision for the defocus stimulus ranging from -4.5 D to 0.5 D than the one provided by the monofocal IOL (Fig. 5) [36].

**Fig. 6:**
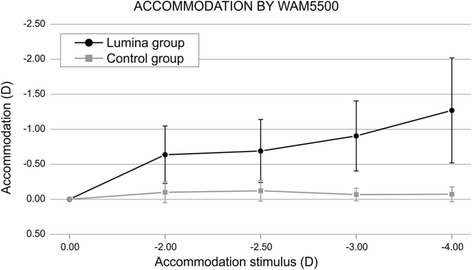
Objective accommodation achieved after implanting the Lumina AIOL. Reprinted with permission from [36]

Additionally, objective accommodation was assessed in that investigation by means of an open field autorefractor WAM-5500 (Grand Seiko, Japan). The WAM allows continuous measurement of the eye refraction while the patient looks at an approaching target through an open field screen [37]. We found that the level of objective accommodation for the Lumina group was statistically significantly better than the monofocal IOL group for the stimulus corresponding to -2.50, -3.00, -3.50 and -4.00 D (Fig. 6) [36].

**Fig. 7:**
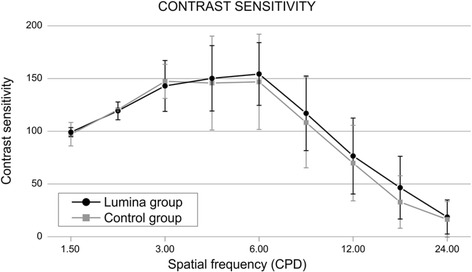
Contrast sensitivity function after Lumina AIOL implantation. Reprinted with permission from [36]

Analysis of the contrast sensitivity function showed no statistically significant differences (*p* >0.05) when comparing the results from the accommodative and monofocal IOL in any of the spatial frequencies analyzed in that investigation (Fig. 7) [36].

Finally, we found no relevant clinical complications during the follow up period. However, it is worth mentioning that the 10 cases required posterior Nd: YAG laser capsulotomy due to posterior capsular opacification [35].

### NuLens AIOL

#### The lens

**Fig. 8:**
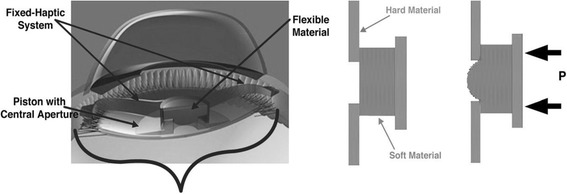
Schematic view of the Nulens AIOL. Reprinted with permission from [38]

The NuLens Dynacurve accommodative IOL (NuLens, Ltd., Herzliya Pituah, Israel) consists of: 1) polymethyl methacrylate (PMMA) haptics that are designed to be placed in the ciliary sulcus; 2) a PMMA anterior reference plane that provides distance vision correction; 3) a small chamber containing a solid silicone gel and 4) a posterior piston with an aperture in the center (Fig. 8) [38].

The mechanism of action of the IOL is as follows: when the ciliary muscle contracts, the forces are transmitted to the piston that induces the gel component to bulge. The optical power of the IOL will increase depending on the magnitude of the silicone bulge due to the contraction of the ciliary muscle (Fig. 8) [38].

In relation to the surgical technique, the IOL must be implanted through a limbal incision of approximately 9 mm in length.

#### Clinical outcomes after NuLens IOL implantation

A clinical study in which 10 patients were implanted with the NuLens and followed during a period of 12 months was conducted. All the patients were diagnosed with cataract and age macular degeneration, thus an adequate assessment of the visual acuity was limited due to macular disease [38]. Nevertheless, regarding the uncorrected near vision a significant increase in the mean number of Jaeger rows that the patient could read increased from preoperatively 1 line to postoperatively 3.8 lines. The mean corrected near vision also showed a slight improvement with a mean gain of 0.7 Jaeger lines [38].

**Fig. 9:**
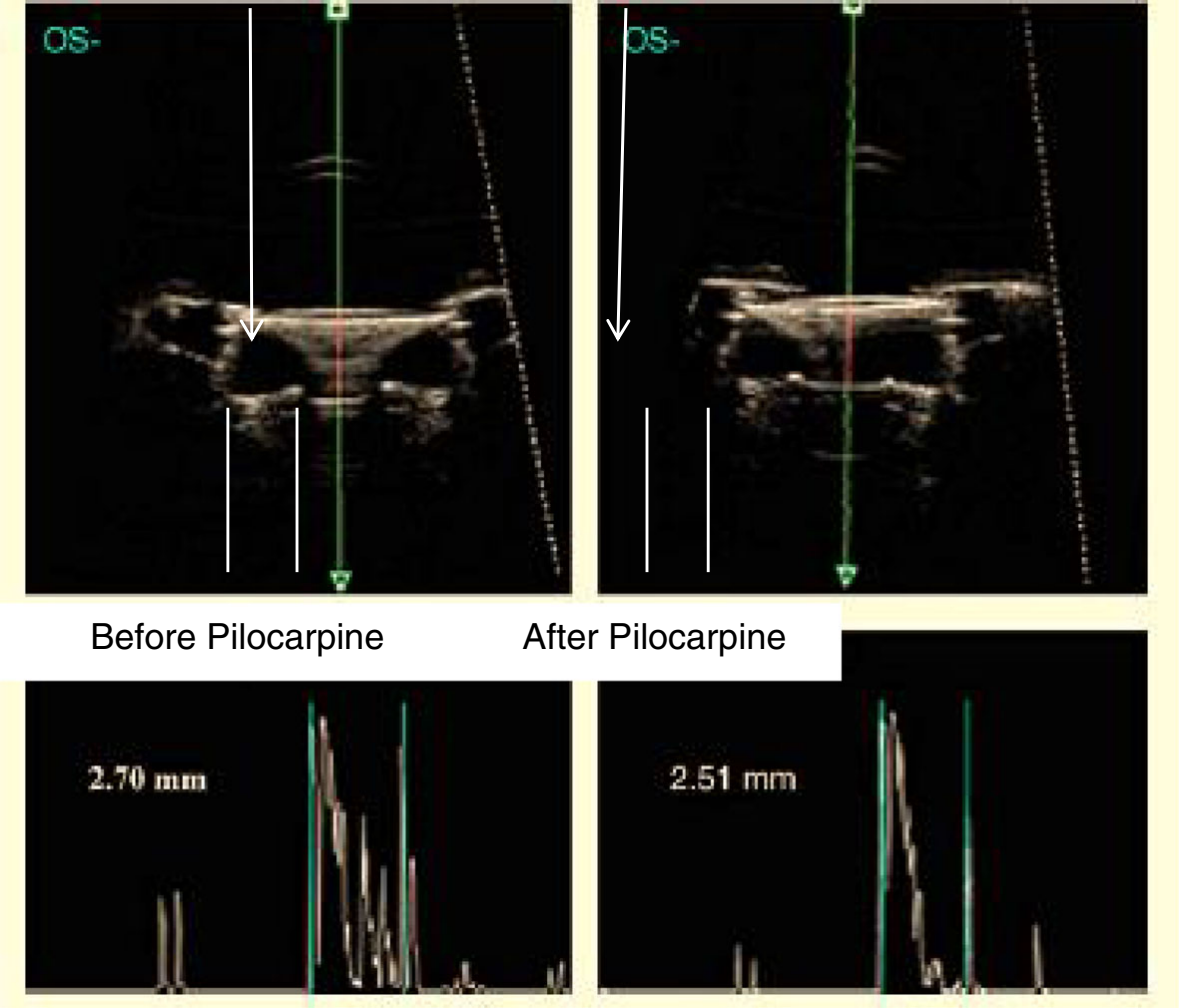
UBM image of the NuLens AIOL showing the cross-sectional movement of the IOL after pilocarpine instillation. Reprinted with permission from [38]

In that study, movement of the IOL was assessed by means of ultrasound biomicroscopy (UBM). Specifically, cross-section movement of the IOL before and after instillation of pilocarpine was evaluated. After contraction of the ciliary muscle induced by the pilocarpine, a bulge of 200 microns in the lens was observed in comparison with the relaxed state 3 months after implantation of the IOL (Fig. 9).

The above-mentioned outcomes with the NuLens IOL were reported in a clinical trial. It is also worth noting that there were 2 serious adverse events observed during the follow up: one posterior synechiae and a capsulorhexis edge capture by the haptic. Both adverse events were resolved after a minor intervention. A large reduction of the endothelial cell count was also found at 3 months after IOL implantation that steadily stabilized over time with no significant change from the 6 to 12 months follow up period. Finally, there was a 60% rate of posterior capsular opacification during the follow up period, which were successfully treated with Nd: YAG laser capsulotomy [38].

### WIOL-CF AIOL

#### The lens

The Wichterle intraocular continuous focus lens (WIOL-CF) (Medicem, Kamenné Žehrovice, Czech Republic) has a polyfocal optic that, in theory, changes shape during the accommodation process [39]. The mechanism by which the lens provides vision at different distances are: 1) polyfocality, which provides high depth of focus due to a hyperbolic optic design; 2) pseudoaccommodation, enabled by a combination of polyfocality and pupillary reflex and 3) accommodation, resulting from a deformation of the lens secondary to a ciliary body contraction that induces an increase on the thickness and a reduction of both anterior and posterior radii of the lens. The lens material is a negatively charged hydrogel from a methacrylic copolymer with a water content of 42%. It has a large diameter optic of 8.6-8.9 mm with a posterior hyperbolic surface that resembles the crystalline lens. Another characteristic of this IOL is that it has no haptics. The hyperbolic posterior surface of the IOL provides infinite foci. The refractive power of the lens decreases from the center to the periphery as does the thickness of the lens that changes from 1.7 mm in the center to 0.8 mm in the periphery [39].

In relation to the surgical technique, the lens can be implanted through a 2.5 to 2.8 mm corneal incision after standard phacoemulsification cataract procedure.

#### Clinical outcomes with the WIOL

In a recent study, data from six different clinical centers taken from the Czech national observational registry with respect to clinical outcomes of patients implanted with the WIOL were evaluated [39]. In that study, 48 patients bilaterally implanted with the WIOL-CF after cataract surgery were assessed during a follow up period of six months. Mean age of the population under analysis was 65 years [39].

In the aforementioned study authors report a mean monocular uncorrected distance visual acuity of 0.07 in LogMAR notation, mean monocular uncorrected near visual acuity of 0.32 and a binocular distance corrected near of 0.26 after six months of follow up [39].

In that study, subjective patient satisfaction was also evaluated by means of a questionnaire. More than 90% of the patients answered satisfied while 8.3% of the patients were unsatisfied. With regards to the wearing of reading glasses, almost half of the population in the study, specifically 47.9% of the patients, did not use reading glasses. On the other hand, 39.6% of the patients used reading glasses occasionally and 12.5% used reading glasses regularly. In relation to photic phenomena, 50% of the patients did not refer to any light phenomena, while 42.9% experienced either halo or glare, but were not severe. Three patients (6.2%) refer to having severe and disturbing photic phenomena [39].

## Discussion

The topic of accommodative intraocular lenses has attracted the attention of a number of ophthalmic surgeons and clinicians as well as the ophthalmic industry. Cataract surgery as a process will not be finished until full restoration of accommodation is accomplished following lens removal with the implantation of an intraocular lens that restores the physiological capability of the human eye to bring a monofocal clear image at different distances. On the contrary to intraocular lenses that are available today, monofocal images are physiologically normal for the human brain and the neuroadaptation that is required for the purpose of the postoperative comfortable use of a multifocal image is not necessary [40]. A confounding factor in the development of the AIOLs used in the past has been the contradictory and many times controversial and commercially biased information about their outcomes. Contradictory information about the performance of some AIOLs models, such as the Crystalens [18–20, 29], with an even more contradictory behavior inside the capsular bag [32] have added further confusion and even discredit to the use of such lenses. The emerging models of AIOLs should solve this controversy by providing sustainable and reliable evidence-based information obtained from well designed and properly performed clinical investigations, including adequate examination methods to explore near vision based in homologated optometrical methods that could detect their performance regarding real accommodation and differentiate them from optical pseudoaccommodation. Once this information is available, the real future perspective of AIOLs will be brilliant and surgeons will be interested again in this challenging topic. The appearance of effective models of widely commercially available AIOLs will be the endpoint of the development and clinical use of the multifocal lenses available today. Surgeons and patients will immediately switch to the use of IOLs based in physiological methods for near vision restoration.

An important issue to consider for the further development of AIOLs is whether such lenses should be placed inside or outside the capsular bag [32]. Recent published primate experimental evidence has demonstrated the limitations of intracapsular IOL support and the advantages of the ciliary sulcus as the most probable preferred location for AIOLs [32], a fact demonstrated by further clinical evidence [36, 38].

## Conclusions

To summarize, based on the information provided in this review, at the present moment, AIOLs are still a developing topic. Implantation inside the capsular bag does not seem to be the most successful approach. The sulcus is probably the best location for the newest generation of AIOLs. Further properly performed clinical research has to confirm those models that are under development today.
